# Meet the Section Editor

**DOI:** 10.2174/1570159X2111230804103519

**Published:** 2023-09-01

**Authors:** Alessandro Pezzini

**Affiliations:** 1 Department of Clinical and Experimental Sciences, Neurology Clinic, University of Brescia, Brescia, Italy

Alessandro Pezzini graduated cum laude in Medicine and Surgery from the University of Brescia, Italy in 1995. In 2002, he became a specialist in Neurology, cum laude, at the same University. He was Research Fellow at the University of Heidelberg in 2001-2002 and then at the University of Munich, Germany, in 2004. In 2007, he was appointed Assistant Professor of Neurology at the University of Brescia in 2010, and Associate Professor in 2015. He is a stroke neurologist at the Neurology Clinic, University of Brescia, Italy. He is member of the Editorial Board of 15 international journals, has served as peer reviewer to over 50 international journals. He is also a member of the Italian Neurological Association (SIN), the World Stroke Organization (WSO), and Fellow of the European Stroke Organization (FESO). Dr. Pezzini is actively involved in international research projects on cervical artery dissections and cerebral non-atherosclerotic vasculopathies, heart-brain interactions, acute ischemic stroke treatment, and intracerebral hemorrhage. He is also a founding member of the Cervical Artery Dissection and Ischemic Stroke Patients (CADISP) network, and the multicentric project Reversible Cerebral Vasoconstriction Syndrome International collaborative study (REVERCE). He authored or co-authored more than 225 papers in peer-review journals, 1 monograph and 14 book chapters.
